# Solid-Contact Potentiometric Sensors Based on Stimulus-Responsive Imprinted Polymers for Reversible Detection of Neutral Dopamine

**DOI:** 10.3390/polym12061406

**Published:** 2020-06-23

**Authors:** Ayman H. Kamel, Abd El-Galil E. Amr, Nashwa H. Ashmawy, Hoda R. Galal, Mohamed A. Al-Omar, Ahmed Y.A. Sayed

**Affiliations:** 1 Department of Chemistry, Faculty of Science, Ain Shams University, Cairo 11566, Egypt; ahkamel76@sci.asu.edu.eg (A.H.K.); nashwastar20@yahoo.com (N.H.A.); 2 Pharmaceutical Chemistry Department, Drug Exploration & Development Chair (DEDC), College of Pharmacy, King Saud University, Riyadh 11451, Saudi Arabia; malomar1@ksu.edu.sa (M.A.A.O.); ahmedyahia009@gmail.com (A.Y.A.S.); 3 Applied Organic Chemistry Department, National Research Center, Dokki 12622, Giza, Egypt; 4 Inorganic Chemistry Department, National Research Center, Dokki 12622, Giza, Egypt; hrgalal@hotmail.com

**Keywords:** potentiometry, stimulus-responsive imprinted polymers (MIPs), solid contact-PEDOT/PSS, dopamine, reversible detection

## Abstract

Herein, we present for the first time a novel potentiometric sensor based on the stimulus-responsive molecularly imprinted polymer (MIP) as a selective receptor for neutral dopamine determination. This smart receptor can change its capabilities to recognize according to external environmental stimuli. Therefore, MIP-binding sites can be regenerated in the polymeric membrane by stimulating with stimulus after each measurement. Based on this effect, reversible detection of the analyte via potentiometric transduction can be achieved. MIPs based on 4-vinylphenylboronic acid as the functional monomer were prepared as the selective receptor. This monomer can successfully bind to dopamine via covalent binding and forming a five- or six-membered cyclic ester in a weakly alkaline aqueous solution. In acidic medium, the produced ester dissociates and regenerates new binding sites in the polymeric membrane. The proposed smart sensor exhibited fast response and good sensitivity towards dopamine with a limit of detection 0.15 µM over the linear range 0.2–10 µM. The selectivity pattern of the proposed ISEs was also evaluated and revealed an enhanced selectivity towards dopamine over several phenolic compounds. Constant-current chronopotentiometry is used for evaluating the short-term potential stability of the proposed ISEs. The obtained results confirm that the stimulus-responsive MIPs provide an attractive way towards reversible MIP-based electrochemical sensors designation.

## 1. Introduction

Dopamine is one of the most critical neurotransmitters belonging to the catecholamine and phenethylamine families. It plays important roles in the systems of the human body, such as its role as a chemical messenger in the central nervous, cardiovascular, hormonal and renal systems [1,2,3]. Changing the level of traceability in dopamine concentration is associated with various neurological diseases such as Alzheimer’s, Parkinson’s, Huntington’s, schizophrenia and epilepsy [4,5,6]. It can also be considered as a clinical medicine in the treatment of cases resulting from heart attacks, trauma, surgery, heart failure, kidney failure and other serious medical conditions. Quantification of dopamine in biologic systems with enhanced sensitivity and good accuracy is very crucial in the clinical diagnosis and in the quality control of dopamine in pharmaceutical products for clinical treatment.

Several analytical methods including high-performance liquid chromatography (HPLC) [7,8], capillary electrophoresis [9], colorimetry [10,11,12], fluorimetry [13,14,15,16], chemiluminescence [17,18] and electrochemical methods [19,20,21] have been reported for dopamine detection. These methods have many disadvantages such as time consuming, requires a complex and trained personal tool, expensive and not suitable for on-site analysis. The main limitation in the use of electrochemical methods for dopamine determination is the potential interference coming from some molecules such as glucose, uric acid and ascorbic acid which may be present with a high concentration in biologic samples [22]. With the hallmarks of high sensitivity, selectivity, easy operation, and a variety of probes available, potentiometric sensors are a good alternative to dopamine detection. Ion-selective electrodes (ISEs) that contain selectivity carriers (i.e., ionophores) have been used widely to detect different types of ions in medical, environmental and industrial analyses [23,24,25,26,27,28].

Molecularly imprinted polymers (MIPs) have attracted wide interest as suitable receptors for all organic and biologic types, due to their advantages in molecular recognition capabilities towards organic and biologic targets [23,24,25,26,27]. They are less expensive, easier to produce and more stable compared to their biologic counterparts such as enzymes and antibodies. It can also bind a wide range of analytes with high affinity and enhanced selectivity similar to those enzymes and antibodies. All these features make this type of ion receptors particularly suitable for their use as promising ionophores in manufacturing ISEs. A stimulus can influence on these responsive-polymers and make them have the ability to alter some of their properties, such as molecular structures, surface properties and degeneration behaviors. In such case, these polymers are known as smart polymers because they posse the stimulus-responsive properties and the capability of molecular recognitions [29,30]. The recognition capabilities of these smart polymers can be controlled by external stimuli such as pH change [31], light [32] or temperature [33]. These smart receivers have shown remarkable success in many applications such as drug delivery, biotechnology as well as separation sciences, but their use in the field of electrochemical sensors is still limited [34,35]. Stimulus-responsive MIP has not been applied in potentiometric sensors fabrication. Designing MIPs is a smart and cost-effective approach to address the massive demand for the detection and quantification of dopamine has been presented in the literature [36,37,38,39].

In this work, we are introducing a new reversible potentiometric sensor based on stimulus-responsive MIP for dopamine determination. MIP is synthesized by using vinyl borate as a functional monomer. This functional group can be stimulated by changing the pH of the solution and can covalently bind with dopamine to form a five-membered cyclic ester. Regeneration of the proposed sensor is easily done via changing the solution from alkali to acidic. The sensors were modified with poly (3,4-ethylenedioxythiophene) (PEDOT) as a solid-contact transducer. Insertion of the conducting polymer (PEDOT/PSS) into the selective membrane increased the hydrophobicity and redox capacitance with considerable potential stability, which was tested by and constant-current chronopotentiometry. The sensors were successfully applied for dopamine determination in human serum and pharmaceutical formulations. Using these smart MIPs in conjunction with potentiometric sensors could provide an effective way to achieve selective, sensitive and reversible detection of dopamine.

## 2. Experimental

### 2.1. Chemicals

All aqueous solutions used in this work were prepared using deionized water (conductivity <0.1µS cm^−1^, Millipore Milli-Q Direct-0.3 purification system). Poly (vinyl chloride) (PVC), 2-nitrophenyloctyl ether (o-NPOE), 4-vinylphenylboronic acid (VPBA), benzoyl peroxide (BPO) and ethylene glycol dimethacrylate (EDGMA) were obtained from Fluka AG (Buchs, Switzerland). Dopamine, poly (3,4 ethylenedioxythiophene)/poly-(styrenesulfonate) (PEDOT/PSS), tridodecyl- methyl-ammonium chloride (TDMACl) and tetradodecylammonium tetrakis (4-chlorophenyl) borate (ETH500) were purchased from Sigma Chemicals Co. (St. Louis, MO, USA). Tetrahydrofuran (THF), NaOH, NaCl and HCl were obtained from Acros (New Jersey, US). All other reagents were analytical grade and used as received. All measurements were performed in 10-mM phosphate buffered solution (PBS) buffer of pH 8 under a constant stirring at room temperature.

### 2.2. Apparatus

All potential readings were recorded at room temperature using PXSJ-216 pH/mV meter (INESA Scientific Instrument Co., Ltd, Shangahi, China). Metrohom potentiostat/galvanostat (Autolab, model 204, Herisau, Switzerland) was used for chronopotentiometry measurements. In these measurements, three-electrode cell containing a reference electrode (Ag/AgCl (3-M KCl)) and an auxiliary electrode (platinum wire) was employed. The applied current is ±1 nA in a solution containing 5-µM dopamine.

### 2.3. Man-tailored Biomimics Synthesis

Man-tailored biomimics (MIPs) were prepared using the free radical polymerization reaction. In brief, 3.0 mmol of cross-linked VPBA monomer with 1.0 mmol of dopamine were dissolved in 20 mL acetonitrile–water mixture (1:1). 0.1-M NaOH was used to adjust the pH of this solution mixture to pH 8. 3.0 mmol of EGDMA was mixed with 80 mg of BPO and added to the reaction mixture. Nitrogen gas stream was diffused into the cocktail solution for 20 min for complete removal of dissolved oxygen and then sealed well. The tube was inserted in an oil bath at 70 °C for 24 h for complete polymerization. Non-imprinted polymers (NIPs) particles were prepared after repeating the previous steps in absence of the template molecule. The resulting powders were washed with 0.1-M HCl and water successively, and then dried under vacuum at 60 °C.

### 2.4. Binding Capacity of the Stimulus MIP

To check the binding availability of the prepared pH-stimulus MIP towards dopamine, 50 mg of the prepared MIP was placed into 10 mL 0.5-mM dopamine at pH 8. The mixture was stirred for 1 h at ambient temperature and then centrifuged for 15 min at 6000 rpm. After that, the obtained MIP particles after the centrifugation was washed with de-ionized water and then immersed into 10 mL 0.1-M HCl and left to stir for 1 h at room temperature. The free dopamine concentration was determined by using an UV-Vis spectrometer at the wavelength of 275 nm. This cycle was repeated three times.

### 2.5. Membrane Preparation and Electrode Fabrication

The polymeric membranes were prepared as: 20.3 mg of the pH-stimulus MIP or NIP, 3.5 mg of TDMAC, 15.2 mg of ETH 500, 103.4 mg of PVC and 212.6 mg of *o-*NPOE were dissolved in 3.5 mL THF. The solid-contact ISEs was prepared as follows: (1) Glassy carbon electrode (GCE) was polished with 0.3 µm Al_2_O_3_ slurries and rinsed with water, followed with acetone, and left in ethanol for 10 min. The resulting clean GCE was inserted into a piece of matched PVC tubing at the distal end; (2) 15 µL of PEDOT/PSS solution was introduced to the GC substrate and left to dry till a uniform layer with strong adhesion to the GCE surface was obtained; (3) 100 µL of the membrane cocktail was drop-cast onto the transducer layer and allowed to dry for 2 h. The coated wire electrodes (CWEs) were prepared by repeating the same steps without adding the solid-contact material (PEDOT/PSS).

Electrode regeneration was achieved after each measurement via treating the sensor with 10-mM HCl for 10 min to release out dopamine from the membrane. After that, the sensor was then conditioned in 10-mM PBS buffer of pH 8 for 10 min.

### 2.6. Dopamine Analysis in Real Samples

The applicability of the proposed ISEs was tested in different human serum samples. The samples were collected from healthy volunteers. The serum was diluted 300 times with 10-mM PBS of pH 8.0 before testing. Finally, 9.0 mL dilute serum solutions were transferred into a 20-mL beaker. The sensor was immersed in conjunction with the reference electrode in the test solution for dopamine analysis.

Dopamine was also analyzed using the proposed ISEs in different commercially available drugs: Dopamine Fresenius (200 mg/5 mL, ampoules) (Fresenius Kapi Co., Cairo, Egypt), Dopaminect (1mg/tablet) (Marcyrle Co., Egypt) and Dopaminect (0.5mg/tablet) (Marcyrle Co., Cairo, Egypt).To examine dopamine in tablet formulations, 3 tablets were grounded in an agate mortar. A specified amount of 3 finely mixed powder disks, equivalent to one tablet, was transferred to a 100-mL volumetric flask and dissolved in 20 mL aqueous 10-mM PBS of pH 8.0, sonicated for 45 min. The solution is then supplemented to the mark with a 30-mM phosphate buffer solution at pH 8. Possible measurements of these solutions were carried out and the potential readings were recorded and compared to the constructed calibration plot. The preparation of the ampoule sample was followed by diluting the appropriate amount of the drug with 30-mM phosphate buffer solution at pH 8.

## 3. Results and discussions

### 3.1. Characterization of the Biomimics Particles

**Figure 1:**
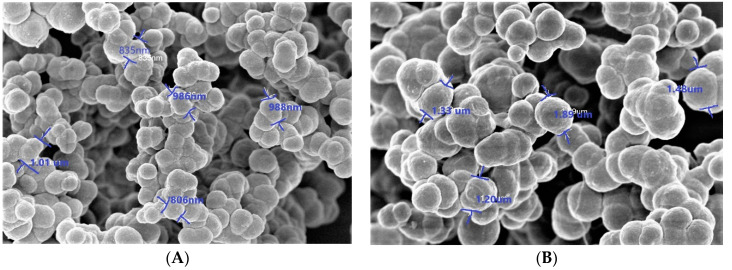
Scanning electron microscope (SEM) images of (**A**) non-imprinted polymers (NIPs) beads and (**B**) washed stimulus-responsive imprinted polymer (MIP)beads.

The morphologic forms of both MIP and NIP surfaces were examined using scanning electron microscope (SEM) (Figure 1). Figure 1A presents a medium uniformity with a spherical shape for the NIP particles with a diameter range from 0.85–1.01 µm. For the MIP particles, the surface morphology presented in Fig. 1B showed irregular beads with a diameter range from 1.2–1.89 µm. The NIP control polymer was observed to have smoother surface and smaller size than the MIP polymer. This can be attributed to the formation of recognition cavities during the synthesis process. The roughness of MIP particles can lead to high surface area than that of NIP and thus MIP can adsorb analytes of interest much better than the NIP. This can confirm the tracing of the printing process that ensures the MIP efficiency as a suitable ionophore for the recognition of dopamine in the presented sensors.

**Figure 2:**
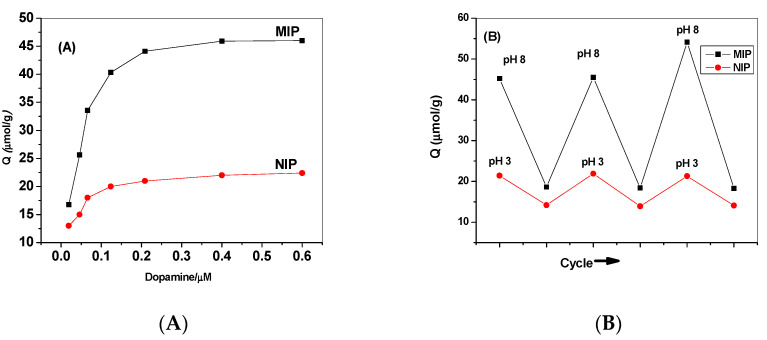
(**A**) Binding isotherms for the prepared polymers; (**B**) binding capacities of both MIP and NIP beads under alkaline (10-mM PBS buffer of pH 7.8) and acid conditions (0.01-M HCl).

The binding availability of the prepared pH-stimulus MIP towards dopamine was evaluated. The binding capacity of either MIP or NIP was calculated according to Equation (1):

Q (µmol/g) = (Ci-C_f_) * Vs *1000 / m (MIP or NIP) (1)

where *Q* is the binding capacity (µmol/g), *Ci* and *C_f_* are the initial and final dopamine concentration (µmol/L), respectively, *Vs* the sample volume (mL) and *m* is the dried mass of the polymer (*g*). As illustrated in Figure 2, the boronic acid–based MIP exhibits a significant change in the binding capacity towards the template dopamine at different pH values than NIP beads. The binding amount of both MIP (45.3 μmol/g) and NIP (21.5 μmol/g) is much higher at pH 8 than that at pH 3.0 [MIP (21.6 μmol/g) and NIP (14.5 μmol/g)], respectively. This can be attributed to the possible covalent interaction that can be formed at pH 8 between the boronic acid groups in the MIP with *Cis*-diol moiety in the dopamine molecule. In this case, a high binding affinity towards dopamine can be observed from the data obtained from MIP and NIP beads. Switching the sample solution to pH 3 hinders the covalent interaction and results in release of the bounded molecules. Repeating the pH-switching cycles reflects an enhanced reversibility of the binding configuration through the uniformed dopamine release and uptake.

### 3.2. Potentiometric Characteristics of the Proposed Sensors

**Figure 3:**
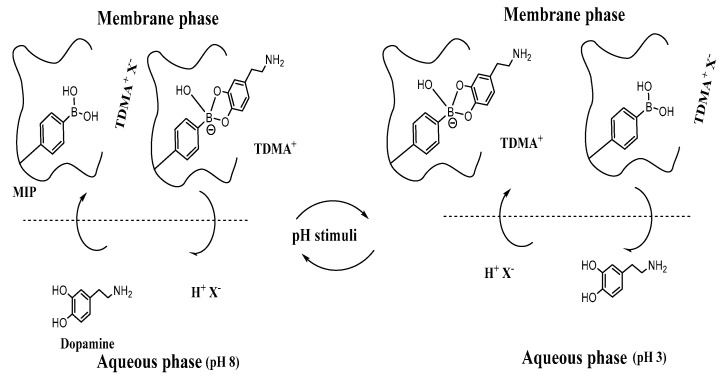
Proposed response and regeneration mechanism.

Under equilibrium conditions, the sensing membrane in ISEs is usually soaked in the ion under investigation and then measured for the Nernstian sensor response. Neutral phenols and their derivatives could reveal strong anionic responses with ion–selective polymeric sensors based on either quaternary ammonium salts or MIPs under near–neutral pH conditions [40,41]. The mechanism of response can be attributed as: (i) Distribution of dopamine molecule between the aqueous phase and the organic phase based on the ion recognition sites in the MIP molecules in the membrane; (ii) The formed complex can interact with quaternary ammonium salts (R^+^) forming MIP/dopamine^−^/R^+^ ion association complex. This can afford H^+^ and X^−^ ions which start moving from the membrane to the aqueous phase revealing the observed anionic response. The observed unexpected anionic response was explained by the net movement of hydrogen ions produced from the phenolic compounds after their interaction with the quaternary ammonium salts in the membrane (Figure 3) [42]. According to these results, we outlined an application of non–classical response in the discovery of dopamine. To guarantee the presence of dopamine in its neutral form, 10-mM phosphate buffer solution of pH 7 was used as a background solution.

**Figure 4:**
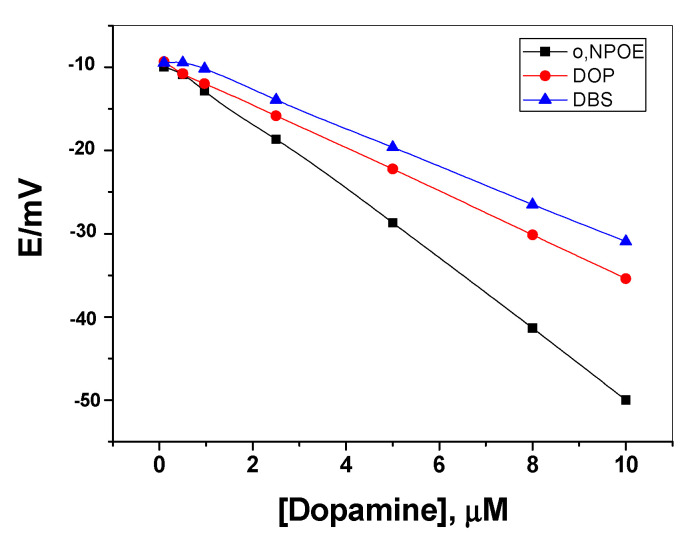
Effect of plasticizers on the membrane response.

**Figure 5:**
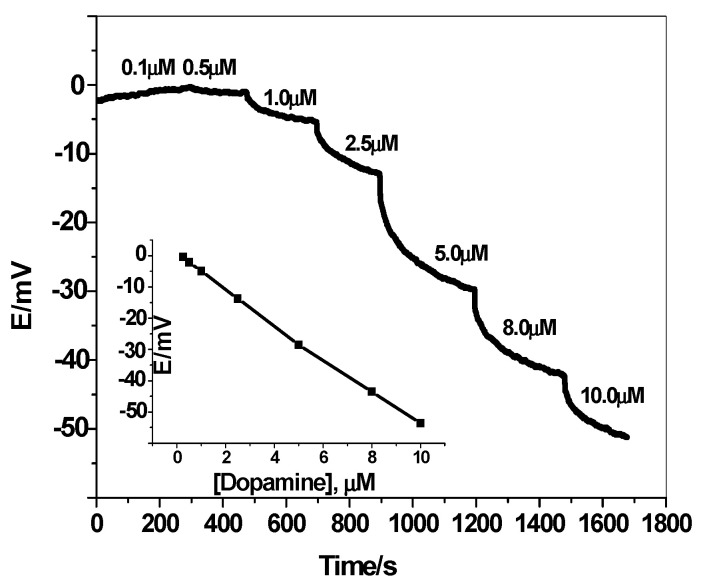
Dynamic potentiometric responses of screen-printed electrodes towards neutral dopamine in 10-mM phosphate buffered solution (PBS)buffer at pH 8. Inset shows the measuring calibration plot for dopamine.

It was reported that the polarity of the membrane solvent not only affects the dissolution of the ionophore in the membrane but also affects the movement of the ion in the membrane phase. So, membrane optimization should be considered in this study. Different plasticizers with different polarities were investigated and their influences on the potential response of the sensing membrane were recorded. As shown in Figure 4, better response behavior and better sensitivity was obtained with the polar plasticizer *o*-NPOE. This can be explained on the basis that dopamine prefers the high polar solvent to be distributed in the sensing membrane. The potential response of the solid-contact sensor towards neutral dopamine is shown in Figure 5. The potential difference between baseline potential and those measured at a specific time (i.e., 120 seconds) was used after the addition of dopamine for quantitative analysis. The results showed a linear relationship between the measured potential and the concentration of neutral dopamine in the range 0.2 to 10 µM and a detection limit of 0.15 µM (3σ). In addition, the presented sensor revealed fast response and stable potential.

The potential change of MIP membrane-based sensor is much larger than those obtained by the NIP at concentration ranging from 0.2 to 10 µM. The calculated sensitivity of NIP membrane-based sensor (1.2 mV/µM) is much lower than that obtained by MIP-membrane based sensor (5.4 mV/µM). This provides the specific recognition interactions between dopamine and the synthesized MIP in the membrane.

**Figure 6:**
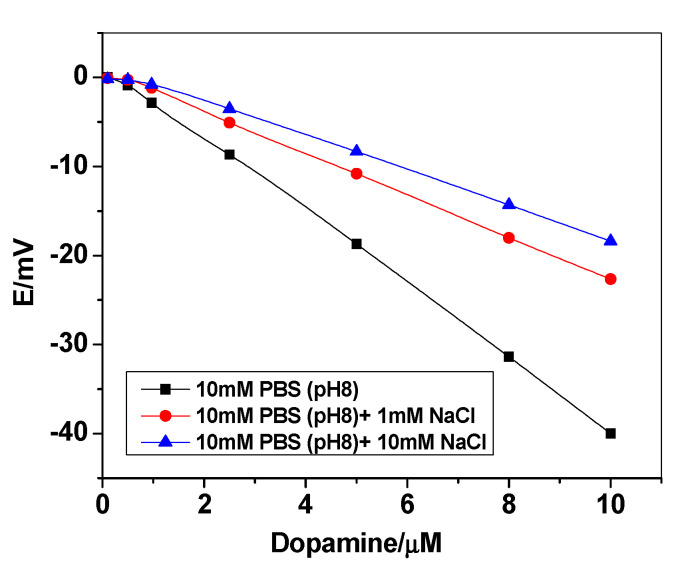
Potentiometric response of the proposed sensor at different chloride concentrations in 10-mM PBS buffer of pH 8.

To optimize the potentiometric anionic response of the proposed sensors, the effect of both X- and H+ in the aqueous phase should be optimized. The anionic response towards neutral phenols is facilitated by a decrease in the concentrations of both X^−^ and H^+^ in the aqueous phase [42]. As shown in Figure 6, a smaller anionic response towards dopamine was observed at higher X^−^ concentrations. All these measurements were carried out at 10-mM PBS of pH 8. This behavior is probably due to the concomitant ejection of H^+^X^−^ from the membrane phase into the aqueous phase could be suppressed by the X^−^ ions in the sample solution.

**Figure 7:**
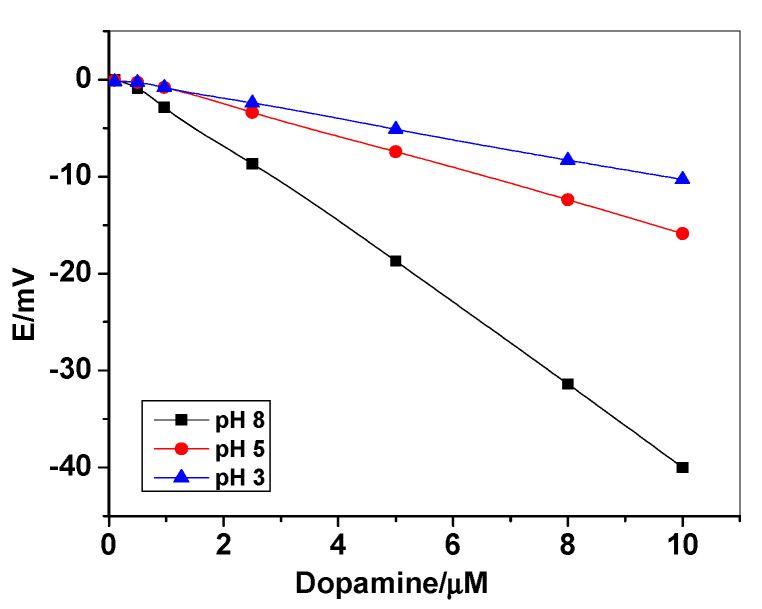
Potentiometric response of the proposed sensor at different pH values in 10-mM PBS buffer +1-mM NaCl.3.3. Sensor Selectivity.

The effect of H^+^ concentration on the potentiometric anionic response was also investigated Figure 7). Three different solutions containing 10-mM NaCl at different pH values (e.g., pH 3, 5 and 8) were taken for studying this effect. It was noticed that the anionic response of the proposed electrode decreases as the concentration of H^+^ ions increases. The chosen sample pH is relatively far from the *pKa* of dopamine (i.e., *pKa* = 8.9) [43] to ensure that dopamine exists in its neutral form. Such similar observation has been obtained by Umezawa et al [42].

**Figure 8:**
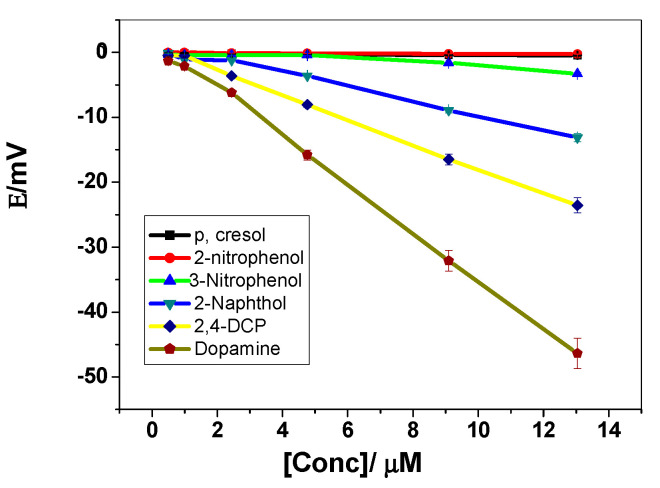
Potentiometric selectivity of MIP membrane-based sensors towards dopamine.

The selectivity coefficient values of the proposed sensors were evaluated using the so called “modified separate solution method (MSSM)” [44]. The potential responses towards dopamine were recorded in a concentration range of 0.5–10 µM (Figure 8). The *pKa* values for phenol derivatives used in selectivity measurements lie in the range 7.8–10.5. Hence, pH 8 is the selected value to ensure the presence of un-dissociated forms of these compounds. Experiments have shown that the selectivity arrangement of the MIP based sensor is dopamine > 2,4-dichlorophenol > 2-naphthol > 3-nitrophenol > 2-nitrophenol >*p*-cresol. The selectivity order of these neutral phenols reflects their acidity and lipophilicity [42]. As the acidity and lipophilicity increases, the anionic response increases. Partition coefficients and acid dissociation constants of dopamine, 2,4-dichlorophenol, 2-naphthol, 3-nitrophenol, 2-nitrophenol and p-cresol are and -0.92, 3.06, 2.7, 2.0, 1.79 and 1.94 and 8.9, 7.89, 9.5, 8.3, 7.23 and 10.3, respectively [43]. In addition to the above-mentioned studied interfering phenols, the effect of urate and ascorbic acid on the potentiometric response of the suggested sensor is also studied. These two compounds are among the substances that interfere during the measurement of dopamine due to their presence in biologic fluids. The sensor does not reveal any interfering effect for these two compounds.

### 3.3. Constant-Current Chronopotentiometry Measurements

**Figure 9:**
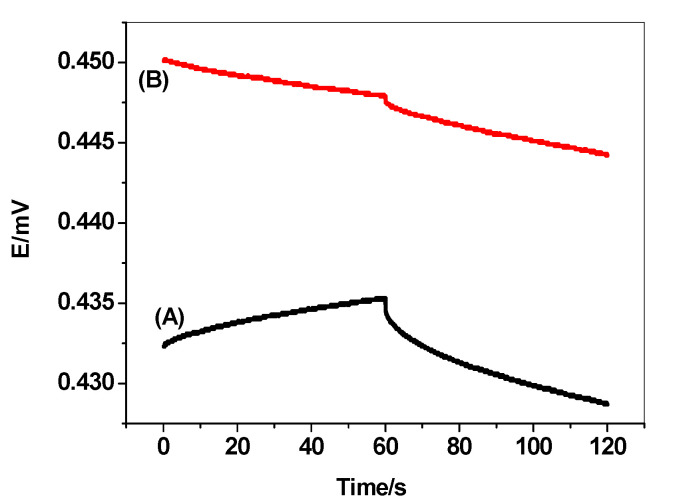
Measurements for dopamine membrane sensor (A) without and (B) with poly (3,4 ethylenedioxythiophene)/poly-(styrenesulfonate) (PEDOT/PSS).

The potential stability of the solid-contact sensors using PEDOT/PSS is critically evaluated by using constant-current chronopotentiometry [45]. Typical chronpotentiogram for the sensors in absence and presence of PEDOT/PSS is shown in Figure 9. The potential drift *(ΔE/Δt*) of the sensors in absence and presence of PEDOT/PSS layer is 82.1 ± 3.1 μV/s (n = 3) and (40.2 ± 1.5 μV/s) (n = 3), respectively. This reflects that insertion of PEDOT/PSS layer between the membrane and the electronic substrate enhances the high potential stability of the electrodes. Due to the ion-to electron transduction process, there is a clear relationship between the short-term potential stability of the sensor and the double layer capacitance of the solid-contact used. The redox capacitance for the proposed electrodes was calculated to be 12.3 ± 0.8 and 25.5 ± 1.3 μF in absence and presence of PEDOT/PSS, respectively.

### 3.4. Dopamine Quantification

The applicability of the proposed ISEs for dopamine determination was checked in some pharmaceutical formulations and human serum samples using the standard addition method. The human samples were diluted to avoid the effect of the matrix. The diluted sample mentioned above was spiked with a certain amount of the three analytes and then analyzed by the standard addition method. The analytical results were shown in (Table 1) indicating minimal interference effect due to the matrix. The recoveries of the spiked sample were found in the range of 96%–101.2%.

Dopamine was also determined in commercially available medicines collected from the domestic market using the standard addition method.The results obtained with measured recovery for each drug are presented in Table 2. A measured accuracy between 94% and 104% indicates that the proposed method for determining dopamine using the displayed electrode is appropriate for routine pharmaceutical analysis. It can be seen that the results from the proposed method were in good agreement with those from labeled amounts (percentage error less than ±5% and the paired *t-*test at 95% below *t_crit_* = 4.3). The results obtained from the proposed potentiometric method are also compared to the method of HPLC from British pharmacopeia, 2009 [46]. From *F*-tests, the results emphasize that there are no significant differences between the results of two methods and revealed the applicability of the proposed sensor as a novel method for the determination of dopamine.

## 4. Conclusion

A reliable, robust and cost-effective solid-contact ISE based on man-tailored mimics for the potentiometric transduction of neutral dopamine was presented. Sensor manufacturing is based on a combination of the use of PEDOT/PSS and the good adhesion capacity revealed by ETH 500. The MIP particles are dispersed into a PVC membrane and projected onto the glassy carbon electrode. The ISEs displayed extended linear response range 0.2–10 µM, low detection limit 0.15-µM and fast response time (< 10 s).Short-term potential stability was tested by constant-current chronopotentiometry techniques. The presented electrodes revealed good advantages over many of those previously described in terms of durability, ease of manufacture, potential stability, selectivity and accuracy. Advantages and disadvantages of many of the previously suggested potentiometric dopaminesensors are presented for comparison in Table 3. The proposed solid-contact dopamine-sensor was successfully used for trace determination of dopamine in different pharmaceutical formulations and human serum samples. No sample pretreatment is required for dopamine analysis using these proposed ISEs.

## Figures and Tables

**Table 1:** Determination of dopamine in spiked human serum samples.

Sample	Dopamine Added, µM		
	Added	Found *	Recovery,%
**1**	2.5	2.4 ± 0.3	96
**2**	5	4.7 ± 0.5	94
**3**	8	8.1 ± 0.8	101.2

* Average of 5 measurements.

**Table 2:** Dopamine determination in pharmaceutical preparations using the proposed membrane sensor.

Pharmaceutical Product and Source	Nominal Content Is Taken	Found, mg			*t*- Student’s Test	*F*-Test
		Proposed Method	Mean ^a^ (%) ±SD	Reference Method (HPLC) [46]		
Dopamine Fresenius (Fresenius Kapi Co., Egypt)	200 mg/5 mL, ampoules	198.7	99.4 ± 0.7	199.3 ± 0.6	1.6	6.3
Dopaminect (Marcyrle Co., Egypt)	1mg/tablet	0.96	94.0 ± 1.2	0.98 ± 0.02	1.5	5.6
Dopaminect (Marcyrle Co., Egypt)	0.5 mg/tablet	0.52	104.0 ± 0.9	0.47 ± 0.03	2.2	7.3

^a^ Mean of three replicate measurements ± standard deviation (SD), *t-*Student and *F*-test at 95% confidence level values are 4.30 and 19.00, respectively.

**Table 3:** General characteristics of some potentiometric dopamine membrane sensors.

Sensory Element	Linear Range, M	Slope, mV/Decade	Detection Limit, M	Working pH	Ref.
Cobalt oxide (Co_3_O_4_) nanowires	10^−9^–10^−2^	52	10^−9^	5.4	[47]
Imprinted polymer based on N- [3-(dimethylamino) propyl] methacrylamide (DMAPM)	4 × 10^−9^–2 × 10^−5^	NR	NR	7.4	[48]
12-Crown-4-phosphotungstic acid-dopamine 12-Crown-4-tetraphenylborate-dopamine	6 × 10^−4^–10^−2^ 8 × 10^−4^–10^−2^	56.2 53.3	5 × 110^−4^ 6 × 110^−4^	2.2–6	[49]
β-cyclodextrin	3 × 10^−5^ to 10^−1^	56.6	2.2 × 10^−5^	6–8	[50]
Dopamine tetraphenylborate	5 × 110^−5^–1 × 10^−2^	55.02	10^−5^	7	[51]
Acrylic polymer molecularly imprinted	>10^−4^	17	10^−4^	7.3	[52]
Dopamine dipicrylamine	6.8x × 10^−5^–3 × 10^−1^	53.3	4.5 × 10^−5^	2–8	[53]
β-cyclodextrin	5 × 10^−5^–1 × 10^−1^	59	8 × 10^−6^	2–7.5	[54]
Bis(triphenylphosphoranylidene) ammonium- periodate	8 × 10^−3^–2.7 × 10^−1^ g/L	310.1 mV g/L	NR	NR	[55]
3,3’-piperazine-bis(phenylboronic acid) 4-octyloxyphenylboronic acid	3 × 10^−4^–10^−2^ 3 × 10^−3^–10^−2^	56.55 3.5	8 × 10^−5^ 2 × 10^−4^	4.5	[56]
heptakis(2,3,6-tri-o-methyl)-β-cyclodextrin	3 × 10^–5^–1 × 10^–3^	43.8	1.3 × 10^−5^	4.4	[57]
Methacrylic acid based molecularly imprinted polymer	2 × 10^−7^–10^−5^	5.4 mV/µM	1.5 × 10^−7^	7	This work

## References

[1] Lee T., Cai L.X., Lelyveld V.S., Hai A., Jasanoff A. (2014). Molecular-level functional magnetic resonance imaging of dopaminergic signaling. Science.

[2] Zhang M., Yu P., Mao L. (2012). Rational design of surface/interface chemistry for quantitative in vivo monitoring of brain chemistry. Acc. Chem. Res..

[3] Lee J.S., Oh J., Kim S.G., Jang J. (2015). Highly sensitive and selective field-effect-transistor nonenzyme dopamine sensors based on Pt/conducting polymer hybrid nanoparticles. Small.

[4] Dalley J.W., Roiser J.P. (2012). Dopamine, serotonin and impulsivity. Neuroscience.

[5] Remy P., Doder M., Lees A., Turjanski N., Brooks D. (2005). Depression in Parkinson’s disease: Loss of dopamine and noradrenaline innervation in the limbic system. Brain.

[6] Shiner T., Seymour B., Wunderlich K., Hill C., Bhatia K.P., Dayan P., Dolan R.J. (2012). Dopamine and performance in a reinforcement learning task: Evidence from Parkinson’s disease. Brain.

[7] Li N., Guo J., Liu B., Yu Y., Cui H., Mao L., Lin Y. (2009). Determination of monoamine neurotransmitters and their metabolites in a mouse brain microdialysate by coupling high-performance liquid chromatography with gold nanoparticle-initiated chemiluminescence. Anal. Chim. Acta.

[8] Cudjoe E., Pawliszyn J. (2014). Optimization of solid phase microextraction coatings for liquid chromatography mass spectrometry determination of neurotransmitters. J. Chromatogr. A.

[9] Wang A.J., Feng J.J., Dong W.J., Lu Y.H., Li Z.H., Riekkola M.L. (2010). Spermine-graft-dextran non-covalent copolymer as coating material in separation of basic proteins and neurotransmitters by capillary electrophoresis. J. Chromatogr. A.

[10] Wen D., Liu W., Herrmann A.K., Haubold D., Holzschuh M., Simon F., Eychmüller A. (2016). Simple and sensitive colorimetric detection of dopamine based on assembly of cyclodextrin-modified Au nanoparticles. Small.

[11] Wang J., Hu Y., Zhou Q., Hu L., Fu W., Wang Y. (2019). Peroxidase-like activity of metal-organic framework [Cu(PDA)(DMF)] and its application for colorimetric detection of dopamine. ACS Appl. Mater. Interfaces.

[12] Guo M.X., Li Y.F. (2019). Cu(II)-based metal-organic xerogels as a novel nanozyme for colorimetric detection of dopamine. Spectrochim. Acta A.

[13] Abdul Rasheed P., Lee J. (2017). Recent advances in optical detection of dopamine using nanomaterials. Microchim Acta.

[14] Lan M., Zhao S., Wei X., Zhang K., Zhang Z., Wu S., Wang P., Zhang W. (2019). Pyrene-derivatized highly fluorescent carbon dots for the sensitive and selective determination of ferric ions and dopamine. Dyes Pigments..

[15] Xu J., Li Y., Wang L., Huang Y., Liu D., Sun R., Luo J., Sun C. (2015). A facile aptamer-based sensing strategy for dopamine through the fluorescence resonance energy transfer between rhodamine B and gold nanoparticles. Dyes Pigments..

[16] Peng J., Han C.L., Ling J., Liu C.J., Ding Z.T., Cao Q.E. (2018). Selective fluorescence quenching of papain–Au nanoclusters by self-polymerization of dopamine. Luminescence.

[17] Lan Y., Yuan F., Fereja T.H., Wang C., Lou B., Li J., Xu G. (2019). Chemiluminescence of lucigenin/riboflavin and its application for selective and sensitive dopamine detection. Anal. Chem..

[18] Sun Y., Lin Y., Ding C., Sun W., Dai Y., Zhu X., Liu H., Luo C. (2018). An ultrasensitive and ultraselective chemiluminescence aptasensor for dopamine detection based on aptamers modified magnetic mesoporous silica@graphite oxide polymers. Sens. Actuators B.

[19] Liu S., Zhang X., Yu Y., Zou G. (2014). A monochromatic electrochemiluminescence sensing strategy for dopamine with dual-stabilizers-capped CdSe quantum dots as emitters. Anal. Chem..

[20] Zhang M., Li J. (2020). Preparation of porphyrin derivatives and C60 supramolecular assemblies as a sensor for detection of dopamine. Dyes Pigments..

[21] Li Y., Yang L., Peng Z., Huang C., Li Y. (2018). Encapsulating a ruthenium(ii) complex into metal organic frameworks to engender high sensitivity for dopamine electrochemiluminescence detection. Anal. Methods.

[22] Wu L., Feng L., Ren J., Qu X. (2012). Electrochemical detection of dopamine using porphyrin-functionalized graphene. Biosens. Bioelectron..

[23] Kamel A.H., Hassan A.M.E. (2016). Solid Contact Potentiometric Sensors Based on Host-Tailored Molecularly Imprinted Polymers for Creatine Assessment. Int. J. Electrochem. Sci..

[24] El-Naby E.H., Kamel A.H. (2015). Potential transducers based man-tailored biomimetic sensors for selective recognition of dextromethorphan as an antitussive drug. Mater. Sci. Eng. C..

[25] El-Kosasy A., Kamel A.H., Hussin L., Ayad M.F., Fares N. (2018). Mimicking new receptors based on molecular imprinting and their application to potentiometric assessment of 2, 4-dichlorophenol as a food Taint. Food Chem..

[26] Kamel A.H., Jiang X., Li P., Liang R. (2018). A paper-based potentiometric sensing platform based on molecularly imprinted nanobeads for determination of bisphenol A. Anal. Methods..

[27] Kamel A.H., Soror T.Y., Al-Romian F.M. (2012). Graphite Solid-Contact Mepiquat Potentiometric Sensors Based on Molecularly Imprinted Polymers and Their Application to Flow Through Analysis. Anal. Meth..

[28] Hassan S.S.M., Badr I.H.A., Kamel A.H., Mohamed M.S. (2009). A Novel Poly (Vinyl Chloride) Matrix Membrane Sensor for Batch and Flow-injection Determination of Thiocyanate, Cyanide and Some Metal Ions. Anal. Sci..

[29] De las Heras Alarcón C., Pennadam S., Alexander C. (2005). Stimuli responsive polymers for biomedical applications. Chem. Soc. Rev..

[30] Islam M.R., Lu Z., Li X., Sarker A.K., Hu L., Choi P., Li X., Hakobyan N., Serpe M. (2013). Responsive polymers for analytical applications: A review. J. Anal. Chim. Acta.

[31] Li L., Lu Y., Bie Z., Chen H.-Y., Liu Z. (2013). Photolithographic boronate affinity molecular imprinting: A general and facile approach for glycoprotein imprinting. Angew. Chem. Int. Ed..

[32] Renkecz T., Mistlberger G., Pawlak M., Horváth V., Bakker E. (2013). Molecularly imprinted polymer microspheres containing photoswitchable spiropyran-based binding sites. ACS Appl. Mater. Interfaces.

[33] Xu S., Lu H., Zheng X., Chen L. (2013). Stimuli-responsive molecularly imprinted polymers: Versatile functional materials. J. Mater. Chem. C..

[34] Wei Y., Zeng Q., Bai S., Wang M., Wang L. (2017). ACS Appl. Mater. Interfaces..

[35] Li Y., Hong M., Bin Q., Lin Z., Cai Z., Chen G. (2013). Novel composites of multifunctional Fe_3_O_4_@ Au nanofibers for highly efficient glycoprotein imprinting. J. Mater. Chem. B.

[36] Mohanan V.M.A., Kunnummal A.K., Biju V.M.N. (2018). Selective electrochemical detection of dopamine based on molecularly imprinted poly(5-amino 8-hydroxy quinoline) immobilized reduced graphene oxide. J. Mater. Sci..

[37] Li Y., Song H., Zhang L., Zuo P., Ye B., Yao J., Chen W. (2016). Supportless electrochemical sensor based on molecularly imprinted polymer modified nanoporous microrod for determination of dopamine at trace level. Biosens. Bioelectr..

[38] Maouche N., Guergouri M., Gam-Derouich S., Jouini M., Nessark B., Chehimi M.M. (2012). Molecularly imprinted polypyrrole films: Some key parameters for electrochemical picomolar detection of dopamine. J. Electroanal. Chem..

[39] Song W., Chen Y., Xu J., Yang X., Tian T. (2010). Dopamine sensor based on molecularly imprinted electrosynthesized polymers. J. Solid State Electrochem..

[40] Zhang H., Yao R., Wang N., Liang R., Qin W. (2018). Soluble Molecularly Imprinted Polymer-Based Potentiometric Sensor for Determination of Bisphenol AF. Anal. Chem..

[41] Hassan S.S.M., Amr A.E., Elbehery N.H.A., Al-Omar M.A., Kamel A.H. (2019). Non-equilibrium potential responses towards neutral orcinol using all-solid-state potentiometric sensors integrated with molecularly imprinted polymers. Polymers.

[42] Ito T., Radecka H., Tohda K., Odashima K., Umezawa Y. (1998). On the Mechanism of Unexpected Potentiometric Response to Neutral Phenols by Liquid Membranes Based on Quaternary Ammonium Salts-Systematic Experimental and Theoretical Approaches. J. Am. Chem. Soc..

[43] https://Pubchem.ncbi.nlm.nih.gov.

[44] Bakker E. (1997). Determination of Unbiased Selectivity Coefficients of Neutral Carrier-Based Cation-Selective Electrodes. Anal. Chem..

[45] Bobacka J. (1999). Potential stability of all-solid-state ion-selective electrodes using conducting polymers as ionto-electron transducers. Anal. Chem..

[46] Comission B.P. (2009). British Pharmacopoeia.

[47] Elhaga S., Ibupotoa Z.H., Liub X., Nura O., Willander M. (2014). Dopamine wide range detection sensor based on modified Co3O4nanowires electrode. Sens. Actuat. B.

[48] Kajisa T., Li W., Michinobu T., Sakata T. (2018). Well-designed dopamine-imprinted polymer interface for selective and quantitative dopamine detection among catecholamines using a potentiometric biosensor. Biosens. Bioelectr..

[49] Othman A.M., Rizk N.M.H., El-Shahawi M.S. (2004). Potentiometric determination of dopamine in pharmaceutical preparations by crown ether-PVC membrane sensors. Anal. Sci..

[50] Ma S., He C., Wang Y., Luo Z., Li G. An all-solid-state ion-selective electrode for dopamine determination. Proceedings of the IET Doctoral Forum on Biomedical Engineering, Healthcare, Robotics and Artificial Intelligence 2018 (BRAIN 2018).

[51] Wołyniec E., Wysocka M., Pruszynski M., Kojło A. (2007). Batch and Flow-Injection Determination of Catecholamines Using Ion Selective Electrodes. Instrum. Sci. Technol..

[52] Pesavento M., D’Agostino G., Biesuz R., Alberti G., Profumo A. (2012). Ion Selective Electrode for Dopamine Based on a Molecularly Imprinted Polymer. Electroanalysis.

[53] Kholoshenko N.M., Ryasenskii S.S., Gorelov I.P. (2006). All-solid-state ion-selective electrodes with ion-to-electron transducers for dopamine determination. Pharm. Chem. J..

[54] Lima J.L.F.C., Montenegro M.C.B.S.M. (1999). Dopamine Ion-Selective Electrode for Potentiometry in Pharmaceutical Preparations. Mikrochim. Acta.

[55] Montenegro M.C.B.S.M., Sales M.G.F. (2000). Flow-Injection Analysis of Dopamine in Injections with a Periodate-Selective Electrode. J. Pharm. Sci..

[56] Durka M., Durka K., Adamczyk-Wozniak A., Wróblewski W. (2019). Dopamine/2-Phenylethylamine Sensitivity of Ion-Selective Electrodes Based on Bifunctional-Symmetrical Boron Receptors. Sensors.

[57] Tanji Y., Wei Q. (2013). Potentiometric Determination of Dopamine Using a Solid-Contact Polymeric Membrane Ion-Selective Electrode. Sensor Lett..

